# Hemodynamic Changes in Left Anterior Descending Coronary Artery and Anterior Interventricular Vein during Right Ventricular Apical Pacing: A Doppler Ultrasound Study in Open Chest Beagles

**DOI:** 10.1371/journal.pone.0067196

**Published:** 2013-06-25

**Authors:** Jing Lu, Wenhua Li, Ying Fu, Bin Long, Jie Shen, Li Su, Lixue Yin

**Affiliations:** Department of Cardiovascular Ultrasound and Non-invasive Cardiology, Sichuan Academy of Medical Sciences and Sichuan Provincial People's Hospital, Chengdu, Sichuan, China; Scuola Superiore Sant'Anna, Italy

## Abstract

**Objective:**

The aim of this study was to quantify the effects of right ventricular apical pacing (RVAP) on hemodynamics in left anterior descending coronary artery (LAD) and anterior interventricular vein (AIV) contrast to baseline condition in open chest beagles using Doppler ultrasound imaging.

**Methods:**

In 6 anesthetized open chest beagles, the spectral Doppler waveforms of the middle segmental LAD and the AIV were acquired with a 5 MHz linear array transducer at baseline condition and during RVAP. The aortic pressure-time curves were recorded synchronously. The Doppler hemodynamic parameters of the LAD and AIV at both states were derived and compared.

**Results:**

The spectral Doppler waveforms of the LAD had a principal diastolic positive wave (Dp), which heelled by a momentary negative wave and a positive wave during early systole at baseline condition. During RVAP, an additional negative wave appeared in the LAD at late systole. The duration of the Dp shortened (227.83±12.16 ms vs 188.50±8.97 ms, *P*<0.001), and the acceleration of the Dp decreased (11.85±2.22 m/s^2^ vs 3.54±0.42 m/s^2^, *P*<0.001). The spectral Doppler waveforms of the AIV only had a principal positive wave (Sp) at baseline condition, but an additional diastolic negative wave appeared during RVAP. The duration of the Sp shortened (242.99±7.98 ms vs 215.38±15.44 ms, *P*<0.001), and the acceleration of the Sp decreased (9.61±1.93 m/s^2^ vs 1.01±0.11 m/s^2^, *P*<0.001).

**Conclusions:**

Obvious hemodynamic changes in the LAD and AIV during RVAP were observed, and these abnormal flow patterns in epicardial coronary arteries and vena coronaria may be sensitive and important hints of the disturbed cardiac electrical and mechanical activity sequences.

## Introduction

Cardiac pacing is one of the most important medical innovations of the 20th century, which is poised to help millions of patients worldwide to live better [1]. Right ventricular apex became the standard placement of the ventricular lead because of easy accessibility and lead stability [2]. Just as a sword with two blades, right ventricular apical pacing (RVAP) was heralded as one of the life-saving treatments for patients with brady-arrhythmias in one hand; at the same time, compelling evidences about its unphysiologic harmful effects on myocardial mechanics and intracardiac hemodynamics were presented in another hand [2], [3].

Healthy heart has perfectly electromechanical synchrony, and the coronary artery and vena coronaria must have its homologous unique blood flow patterns. RVAP will definitely induce ventricular asynchrony, which has been confirmed as an unphysiologic pacing mode. We hypothesize that: 1) Hemodynamic characteristics of the left anterior descending coronary artery (LAD) and the anterior interventricular vein (AIV) in healthy hearts may be important reflections of physiological cardiac electrical and mechanical activity sequences. 2) Hemodynamics in the LAD and the AIV might be changed by RVAP, and such changes could be taken as a new window for insight the disturbed cardiac electrical and mechanical activity sequences.

Blood stream in the LAD of open chest dogs can be imaged clearly by Color Doppler ultrasound with linear array transducer [4]. But the relations between the blood flow patterns in LAD and AIV with the natural electrical activity sequences and the traditional cardiac pacing modes have not yet been established with this technique. The aim of this study was to quantify the effects of RVAP on hemodynamics of the LAD and the AIV contrast to baseline condition in open chest beagles using Doppler ultrasound imaging.

## Materials and Methods

### Ethics Statement

All animals involved received humane care in compliance with accredited facilities of Chinese Association for Accreditation of Laboratory Animal Care. The studies and animal care protocols were reviewed and approved by the Institutional Care and Animal Research Committee of Sichuan Provincial People's Hospital. This study was carried out in strict accordance with the recommendations in the Guide for the Care and Use of Laboratory Animals of the Ethics Committee of Sichuan Provincial People's Hospital. Each surgery was performed under general anesthesia, and all efforts were made to minimize suffering.

### General Preparation

Six healthy beagles (3 males and 3 females; 12.3–17.2 kg, mean weight 15.22±1.85 kg) were provided by Experimental Animal Institute of Sichuan Academy of Medical Sciences. Preoperative 12 hours fasting was applied to each animal. Intramuscular injection of atropine 0.5 mg and aminazine 50 mg were used for sedation. And thirty minutes later, ketamine 100 mg and fentanyl 100 mg were given intramuscularly for anesthesia. Anaesthetizd beagles were placed in a supine position. A polyvinyl cannula was inserted into the left femoral vein for drug and fluid administration. Slow intravenous infusion of saline maintained hydration throughout surgery, and anesthesia was maintained using continuous intravenous infusion of ketamine (500 mg/h) and fentanyl (500 mg/h). Mechanical ventilation was used via an orotracheal tube with 40–60% O_2_, ventilation frequency: 20 bpm, tidal volume: 10–15 ml/kg/min (IE 902-C ventilator; Ruideyigeer Co, China). Supplemental doses of anesthetics were given throughout the experiment to suppress the medial ocular reflex. Arterial blood gases and pH were kept stable by adjusting ventilation or bicarbonate infusion. All the sheaths and catheters were washed and filled using heparin sodium solution before insertion. The heart was exposed adequately by a lateral thoracotomy and suspended in a pericardial cradle. Under the monitoring of gray-scale echocardiography, a 7F Millar catheter tip was introduced into the aortic root via left carotid artery, with electrocardiography (ECG) and pressure monitoring (Lead 2000; Jinjiang Electronic Co, China). A bipolar pacing electrode lead was implanted directly into the subepicardium of right ventricular apical anterior wall after baseline data collection had been completed. RVAP was performed with fixed pacing rate at 160 bpm, and with fixed pulsed stimulating voltage at 2 volt (Lead 2000; Jinjiang Electronic Co, China).

### Ultrasound Imaging

**Figure 1:**
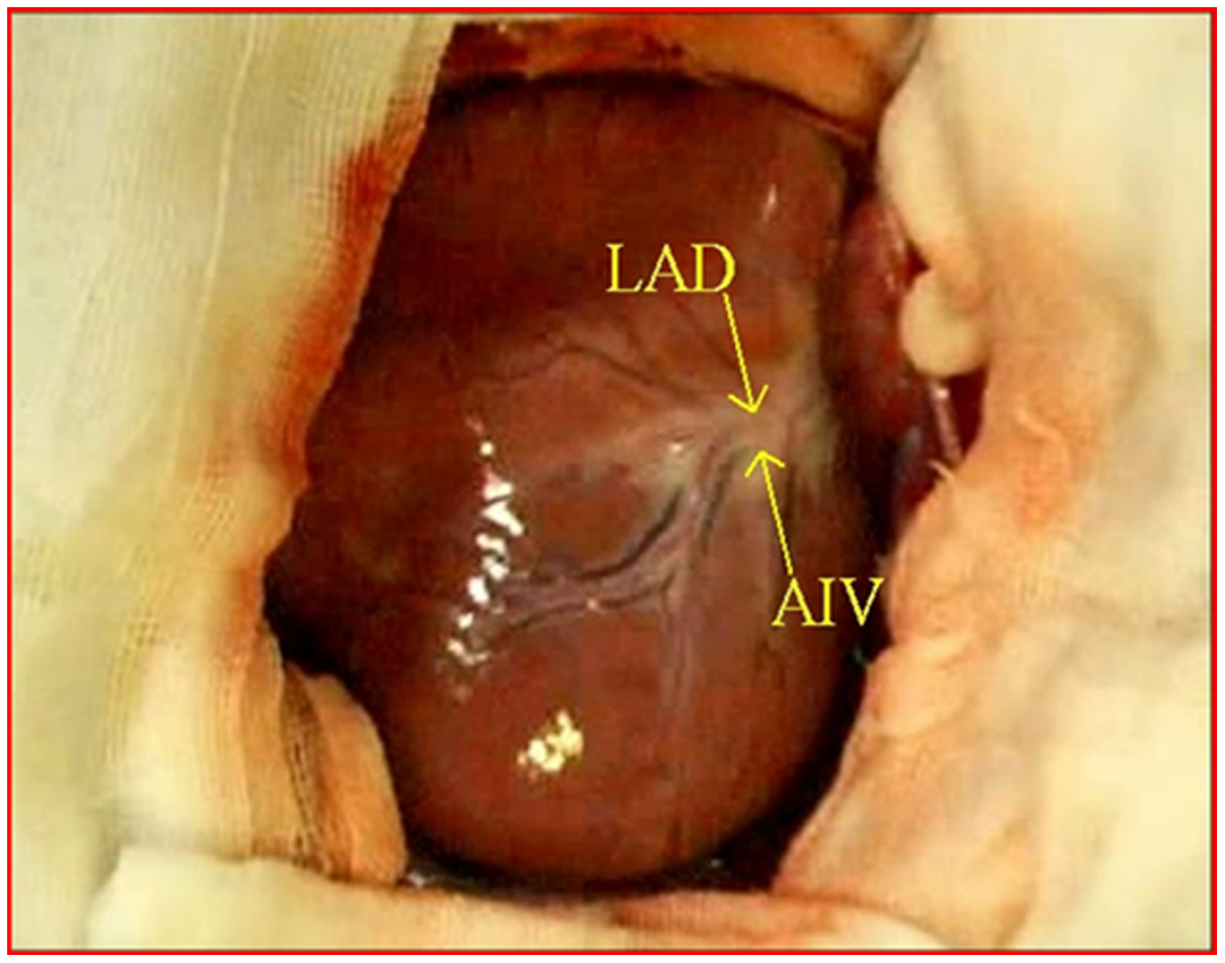
The middle segments of the LAD and the AIV. LAD = left anterior descending coronary artery; AIV = anterior interventricular vein.

Commercially available ultrasonic equipment (Prosound α10; Aloka Co, Japan) with a 1–5 MHz phased array cardiac transducer (UST-52101) and a 4–13 MHz linear phased array transducer (UST-5412) was utilized for all beagles ultrasonic imaging. The left ventricle (LV) real-time two-dimensional gray-scale images of standard apical 4-chamber view and standard apical 2-chamber view in 3 consecutive cardiac cycles were acquired with the phased array cardiac transducer and stored both at baseline conditions and during RVAP for measurement of the LV volumetric parameters. For assessment of global LV diastolic function, we acquired and measured LV isovolumetric relaxation time (IVRT), LV early peak filling velocity (E), and early diastolic velocity of mitral annulus (e′). The e′ was the average tissue Doppler velocity of the septal and lateral sides of mitral annulus during early diastole. The septal E/e′ ratio (E/e′) was calculated. The linear phased array transducer was applied to acquire the gray-scale images, the color Doppler flow images, and the spectral Doppler waveforms of the LAD and the AIV at both states. The preset for periphery arteries application was used, and the center frequency of the linear transducer was set at 5 MHz. The middle segments of the LAD and AIV, which just distal to the first diagonal branch, were interrogated in this study (Fig. 1). To maintain image quality, sufficient coupling medium was added into the space between the linear transducer and the epicardium during ultrasound scanning. We acquired the gray-scale images of the LAD and the AIV at its minor axis cut plane, and the real-time images in 3 consecutive cardiac cycles were stored. Then, the sampling box of color Doppler flow imaging was turned on, and its suitable size, position, and steer angle were adjusted properly. The maximal velocity range of the color bar was preset from 17 to 25 cm/s with its baseline at 0 cm/s. To acquire the spectral Doppler waveforms of the LAD and AIV, the transducer was moved with about 90° clockwise rotation from the minor axis cut plane of the LAD and AIV. After viewing the length of the vessel, the pulse-wave Doppler was turned on. The sampling gate was set at 1.5 mm, and the angle between the blood flow and ultrasound beam was optimized and corrected to less than 30°. The stable spectral Doppler waveforms of the LAD and AIV in 3 consecutive cardiac cycles were acquired and stored at both states respectively. At the same time, the aortic pressure-time curves were stored on hard disk synchronously for off-line analysis.

### Data Analysis and Processing

All of the under-mentioned quantitative data were measured 3 times on 3 different cardiac cycles, and the average values were calculated and recorded. All the time parameters at baseline condition were normalized by making linear correction in light of the fixed heart rate of 160 bpm.

LV end diastolic volume (LVEDV), end systolic volume (LVESV), stroke volume (LVSV), ejection fraction (LVEF) and cardiac output (CO) were derived via biplane Simpson rule. The real-time gray-scale images of the LAD and AIV at its minor axis cut plane were retrieved frame by frame until the maximal area of the LAD or the maximal area of the AIV appeared, then the maximal diameter of the LAD (Φ_LAD_) or the maximal diameter of the AIV (Φ_AIV_) was measured on the static images respectively. When we measured the Φ_LAD,_ the interval between the LAD and the AIV was recorded at the same time.

**Figure 2:**
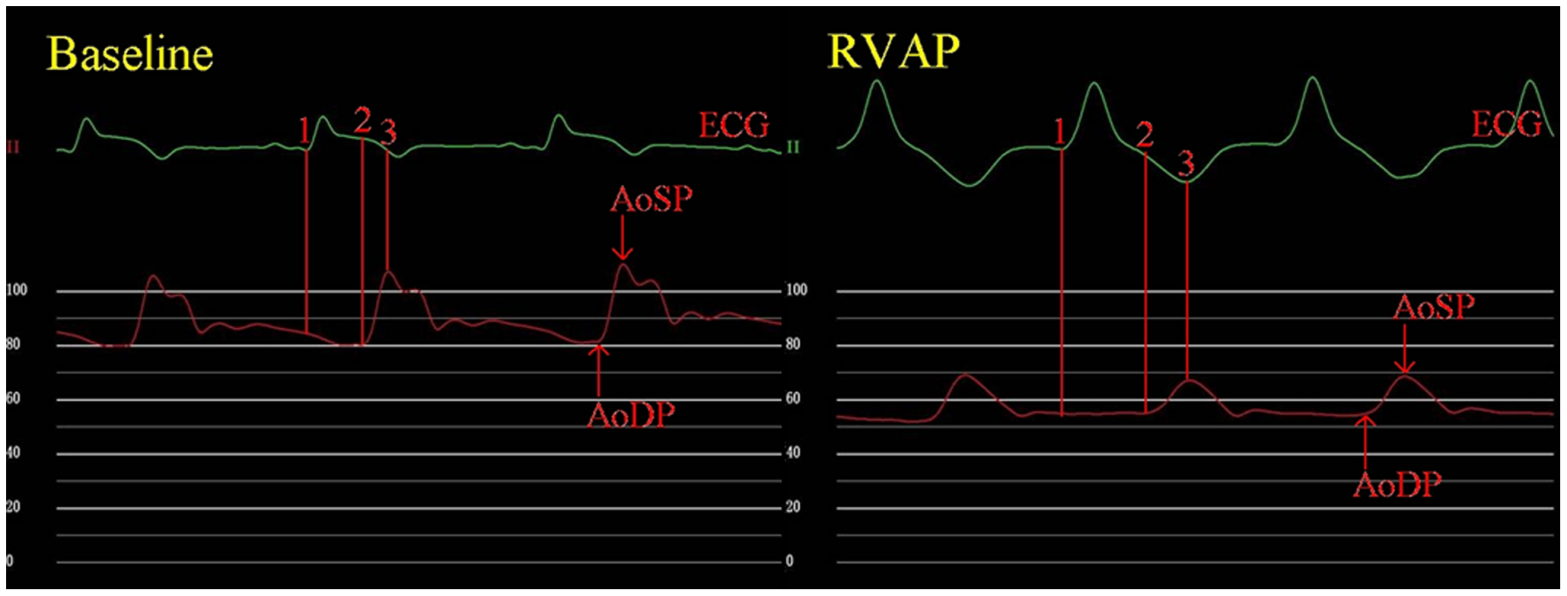
Duration between the line 1 and the line 2 represents the time from the onset of the QRS in the ECG to the measure point of AoDP on the synchronous red aortic pressure-time curve (TAoDP). Duration between the line 1 and the line 3 represents the time from the onset of the QRS in the ECG to the measure point of AoSP on the synchronous red aortic pressure-time curve (TAoSP). Baseline = baseline condition; RVAP = right ventricular apical pacing; AoDP = aortic diastolic pressure; AoSP = aortic systolic pressure.

The aortic diastolic pressure (AoDP) and the aortic systolic pressure (AoSP), and the aortic pressure upstroke time (T) were measured through pressure-time curves stored in electrophysiologic equipment. And the aortic pulse pressure difference (△P) and the mean upstroke velocity of the aortic pressure (△P/T) were calculated: △P = AoSP−AoDP; △P/T = (AoSP−AoDP)/T. We also measured the time from the onset of the QRS (QRS_O_) in the ECG to the measure point of AoDP on the synchronous aortic pressure-time curve (TAoDP) and the time from the QRS_O_ in the ECG to the measure point of AoSP on the synchronous aortic pressure-time curve (TAoSP) (Fig. 2).

Spectral Doppler waveform analysis was performed on the ultrasonic machine. Various Doppler hemodynamic parameters were acquired according to the different spectral Doppler waveforms of the LAD and the AIV at each state.

Two double-blinded examiners repeatedly performed the spectral Doppler waveform analysis for intraobserver and interobserver analysis of the Doppler hemodynamic parameters of the LAD and the AIV. The second observer repeated the measurements 2 weeks after the first time.

### Statistical Analysis

Statistical tests were performed with SPSS 13.0 statistical software (SPSS, Chicago, IL, USA). Quantitative data are expressed as mean ± SD. We tested for data normality using the Shapiro-Wilk test. The cardiac volumetric parameters, the aortic pressure parameters, and the Doppler hemodynamic parameters of the LAD and the AIV were compared between each state respectively using the paired Student *t* test. Correlation analysis was performed between the aortic pressure parameters and the Doppler hemodynamic parameters of the LAD and the AIV. The bias tests for intraobserver and interobserver agreement were compared using paired *t* test. And intraobserver and interobserver variability was assessed by calculating the coefficient of variability. Statistical significance was defined as *P*<0.05. For *P* values <0.000, a value of <0.001 was chosen.

## Results

### Effect of RVAP on the LV Function and the Aortic Pressure

We found statistical significance existing in the LV function parameters and the aortic pressure parameters between each state except for the LVESV and the CO (Table 1). The heart rate increased and the aortic pressure upstroke time lengthened during RVAP (*P*<0.001). The LVEDV, the LVSV, the LVEF, the IVRT, the E, the e′, the E/e′, the AoSP, the AoDP, the △P, and the △P/T decreased during RVAP (*P*<0.01). The TAoDP and the TAoSP lengthened during RVAP (*P*<0.001).

**Table 1:** Comparisons of LV function and the aortic pressure parameters between each state.

Variable	Baseline (n = 6)	RVAP (n = 6)	*t* value	*P* value
HR (beat/min)	132.67±10.33	160.00±0.00	6.483	0.001
LVEDV (ml)	17.89±1.25	16.19±1.17	4.510	0.006
LVESV (ml)	8.04±0.68	8.45±0.71	2.005	0.101
LVSV (ml)	10.51±0.93	10.09±7.76	8.086	0.001
LVEF (%)	55.09±2.08	47.77±2.70	8.989	0.001
CO (ml/min)	1308.86±159.83	1237.89±120.83	1.039	0.346
IVRT (ms)	80.13±7.01	60.64±8.28	21.253	0.001
E (cm/s)	46.08±6.72	33.17±5.60	16.357	0.001
e′ (cm/s)	6.17±0.64	5.83±0.65	2.774	0.039
E/e′	7.46±0.58	5.67±0.62	10.025	0.001
AoDP (mmHg)	73.41±11.42	46.60±9.52	18.155	0.001
AoSP (mmHg)	102.94±10.60	89.00±16.14	25.111	0.001
△P (mmHg)	29.53±2.61	17.71±2.65	12.015	0.001
T (ms)	41.94±1.60	71.97±2.15	32.958	0.001
△P/T (mmHg/s)	704.48±35.07	245.93±35.07	19.086	0.001
TAoDP (ms)	88.12±7.53	134.26±9.79	18.829	0.001
TAoSP (ms)	130.05±6.80	206.24±10.02	24.685	0.001

Baseline = baseline condition; RVAP = right ventricular apical pacing; HR = heart rate; LVEDV = left ventricular end diastolic volume; LVESV = left ventricular end systolic volume; LVSV = left ventricular stroke volume; LVEF = left ventricular ejection fraction; CO = cardiac output; IVRT = left ventricular isovolumetric relaxation time; E = left ventricular early peak filling velocity; e′ = early diastolic velocity of mitral annulus; E/e′ = septal E/e′ ratio; AoDP = aortic diastolic pressure; AoSP = aortic systolic pressure; △P = aortic pulse pressure difference; T = aortic pressure upstroke time; △P/T = mean upstroke velocity of the aortic pressure; TAoDP = time from the onset of the QRS to the measure point of AoDP on the synchronous aortic pressure-time curve; TAoSP = time from the onset of the QRS to the measure point of AoSP on the synchronous aortic pressure-time curve.

Results were expressed as mean ± SD with six data points collected in each analysis. The *t* values and the *P* values were given by comparing the results of the baseline with RVAP using paired Student’s *t* test. Statistical significance was defined as *P*<0.05. For *P* values <0.000, a value of <0.001 was chosen.

### Anatomic Quantification of the LAD and the AIV on the Gray-scale Images

No statistical significance was found between the Φ_LAD_ and the Φ_AIV_ at both states (Baseline: 1.92±0.09 mm vs 1.82±0.10 mm, *t* = 2.253, *P* = 0.074; RVAP: 1.88±0.07 mm vs 1.83±0.07 mm, *t* = 1.550, *P* = 0.182). And no statistical significance was found in the Φ_LAD_, the Φ_AIV_, and the I_LAD-AIV_ between each state (Table 2).

**Table 2:** Comparisons of the anatomic quantification of the LAD and the AIV between each state.

Variable	Baseline (n = 6)	RVAP (n = 6)	*t* value	*P* value
Φ_LAD_ (mm)	1.92±0.09	1.88±0.07	1.379	0.226
Φ_AIV_ (mm)	1.82±0.10	1.83±0.07	0.558	0.601
I_LAD-AIV_ (mm)	2.13±0.27	2.10±0.20	0.566	0.596

Baseline = baseline condition; RVAP = right ventricular apical pacing; LAD = left anterior descending coronary artery; AIV = anterior interventricular vein; Φ_LAD_ = maximal diameter of the LAD; Φ_AIV_ = maximal diameter of the AIV; I_LAD-AIV_ = interval between the LAD and the AIV.

Results were expressed as mean ± SD with six data points collected in each analysis. The *t* values and the *P* values were given by comparing the results of the baseline with RVAP using paired Student’s *t* test. Statistical significance was defined as *P*<0.05. For *P* values <0.000, a value of <0.001 was chosen.

### The Spectral Doppler Waveforms of the LAD and the AIV at both States (Fig. 3)

In all animals, the spectral Doppler waveform characteristics of the LAD were: 1) The LAD at both states had a principal positive wave (Dp) during diastole, and heeled by a momentary negative wave (S1) and a positive wave (S2) during early systole. 2) Transitory cessation flow in the LAD during mid to late systole at the baseline condition. 3) During RVAP, an additional negative wave (S3) appeared at late systole that did not exist in the LAD at baseline condition.

**Figure 3:**
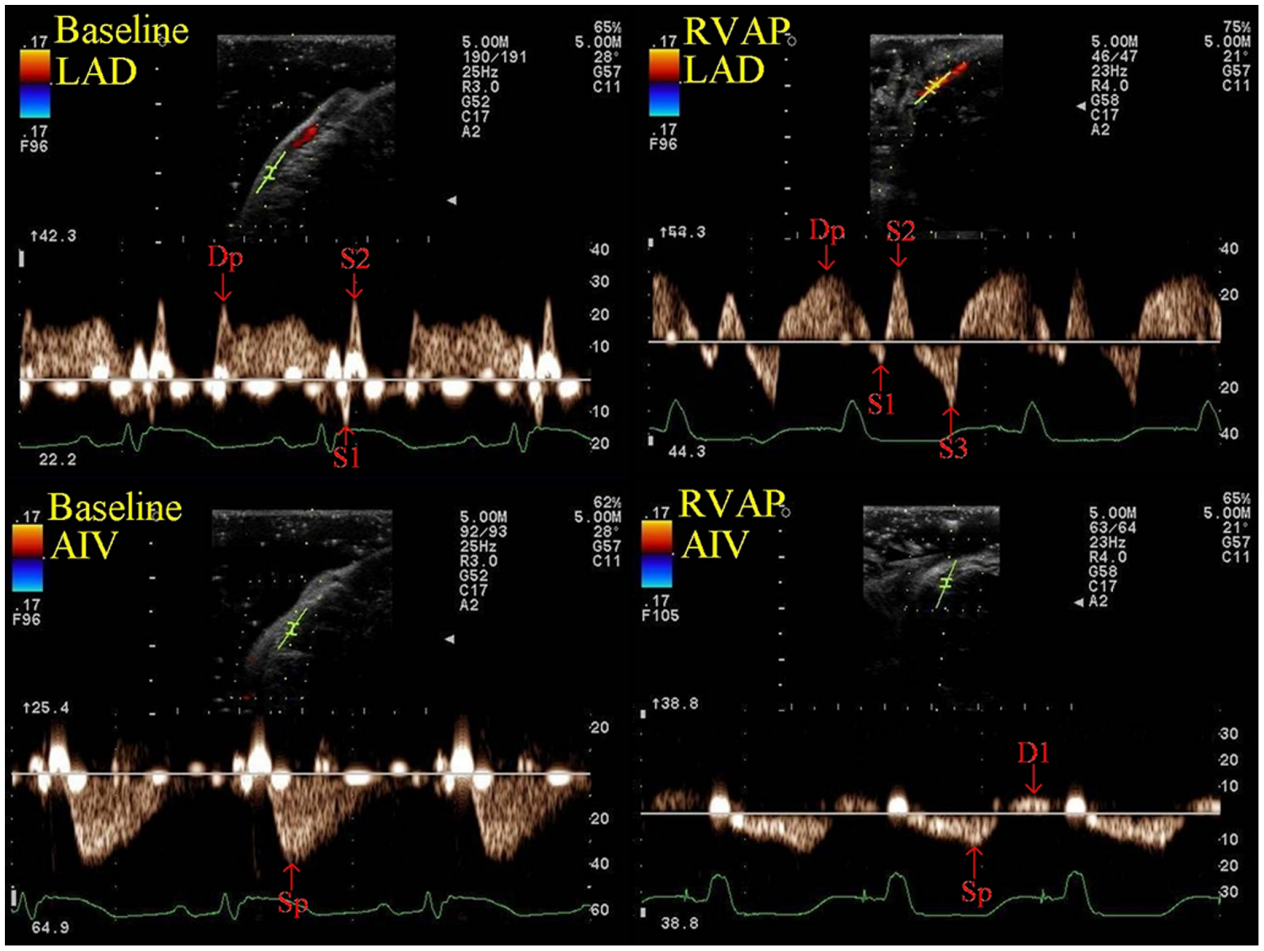
The spectral Doppler waveforms of the middle segment LAD and the AIV at both states in one open chest beagle. The spectral Doppler waveforms of the LAD at baseline condition had a principal positive wave (Dp) during diastole, and heeled by a momentary negative wave (S1) and a positive wave (S2) during early systole, and a transitory cessation flow appeared during mid to late systole (upper row left panel). During RVAP, a negative wave (S3) appeared at late systole that did not exist in the LAD at baseline condition (upper row right panel). The spectral Doppler waveforms of AIV only had a principal positive wave (Sp) at baseline condition, and the Sp appeared at the middle systole and lasting almost the whole diastole, and a momentary cessation flow appeared at the end of diastole (lower row left panel). During RVAP, the Sp of the AIV appeared at the early systole and heeled by a diastolic negative wave (D1) (lower row right panel). Baseline = baseline condition; RVAP = right ventricular apical pacing; LAD = left anterior descending coronary artery; AIV = anterior interventricular vein.

In all animals, the spectral Doppler waveform characteristics of the AIV were: 1) The AIV only had a principal positive wave (Sp) at baseline condition, and the Sp appeared at the middle systole and lasting almost the whole diastole. 2) A momentary cessation flow at the end of diastole in the AIV at baseline condition. 3) During RVAP, the Sp appeared at the early systole and heeled by an additional diastolic negative wave (D1) that did not exist in the AIV at baseline condition.

### Comparisons of Doppler Hemodynamic Parameters of the LAD between each State (Table 3)

Compared between each state: 1) No statistical significance was found in the peak velocity of the Dp, the peak velocity of the S1, and the peak velocity of the S2 between each state. 2) The duration of the Dp shortened, but the duration of the S1 and the duration of the S2 lengthened during RVAP (*P*<0.001). 3) The accelerating time of the Dp lengthened, the acceleration of the Dp and the percentage of the duration of the Dp (D-Dp%) decreased during RVAP (*P*<0.001). The D-Dp% was calculated as the percentage of the duration of the Dp divided by the R-R interval. 4) The time from the QRS_O_ to the onset of the Dp and the time from the QRS_O_ to the peak of the Dp lengthened during RVAP (*P*<0.001). 5) The peak velocity of the S3 in the LAD during RVAP was 24.84±3.01 cm/s, and its duration was 25.06±1.69 ms.

**Table 3:** Comparisons of Doppler hemodynamic parameters of the LAD between each state.

Variable	Baseline (n = 6)	RVAP (n = 6)	*t* value	*P* value
Vp-Dp (cm/s)	28.61±6.53	32.69±5.12	2.191	0.080
D-Dp (ms)	227.83±12.16	188.50±8.97	20.907	0.001
D-Dp % (%)	60.75±3.24	50.27±2.39	20.907	0.001
AT-Dp (ms)	23.96±1.32	92.10±4.19	42.795	0.001
Ac-Dp (m/s^2^)	11.85±2.22	3.54±0.42	10.813	0.001
Vp-S1 (cm/s)	10.71±1.58	10.67±1.37	0.149	0.887
D-S1 (ms)	21.42±1.23	31.38±0.95	27.059	0.001
Vp-S2 (cm/s)	27.26±1.60	26.11±2.02	1.083	0.328
D-S2 (ms)	39.19±0.89	60.17±1.74	31.653	0.001
TODp (ms)	202.40±9.10	241.78±9.13	7.328	0.001
TPDp (ms)	226.36±8.43	333.87±9.41	18.501	0.001

Baseline = baseline condition; RVAP = right ventricular apical pacing; LAD = left anterior descending coronary artery; Vp-Dp = peak velocities of the Dp; D-Dp = duration of the Dp; D-Dp % = percentage of the duration of the Dp divided by the R-R interval; AT-Dp = accelerating time of the Dp; Ac-Dp = acceleration of the Dp; Vp-S1 = peak velocities of the S1; D-S1 = duration of the S1; Vp-S2 = peak velocities of the S2; D-S2 = duration of the S2; TODp = time from the onset of the QRS to the onset of the Dp; TPDp = time from the onset of the QRS to the peak of the Dp.

Results were expressed as mean ± SD with six data points collected in each analysis. The *t* values and the *P* values were given by comparing the results of the baseline with RVAP using paired Student’s *t* test. Statistical significance was defined as *P*<0.05. For *P* values <0.000, a value of <0.001 was chosen.

### Comparisons of Doppler Hemodynamic Parameters of the AIV between each State (Table 4)

Compared between each state: 1) The peak velocity of the Sp in the AIV decreased, and the duration of the Sp shortened and the percentage of the duration of the Sp (D-Sp%) decreased during RVAP (*P*<0.001). The D-Sp% was calculated as the percentage of the duration of the Sp divided by the R-R interval. 2) The time from the QRS_O_ to the onset of the Sp and the time from the QRS_O_ to the peak of the Sp shortened during RVAP (*P*<0.001). 3) The accelerating time of the Sp lengthened, and the acceleration of the Sp decreased during RVAP (*P*<0.001). 4) The peak velocity of the D1 in the AIV during RVAP was 7.22±1.41 cm/s, and its duration was 32.47±2.28 ms.

**Table 4:** Comparisons of Doppler hemodynamic parameters of the AIV between each state.

Variable	Baseline (n = 6)	RVAP (n = 6)	*t* value	*P* value
Vp-Sp (cm/s)	36.47±7.09	13.50±1.71	9.900	0.001
D-Sp (ms)	242.99±7.98	215.38±15.44	7.532	0.001
D-Sp % (%)	64.79±2.13	57.44±4.12	7.532	0.001
AT-Sp (ms)	39.37±11.69	135.54±26.32	12.682	0.001
Ac-Sp (m/s^2^)	9.61±1.93	1.01±0.11	11.272	0.001
TOSp (ms)	103.46±6.52	54.91±7.22	16.522	0.001
TPSp (ms)	141.61±7.74	188.50±10.76	8.210	0.001

Baseline = baseline condition; RVAP = right ventricular apical pacing; AIV = anterior interventricular vein; Vp-Sp = peak velocities of the Sp; D-Sp = duration of the Sp; D-Sp % = percentage of the duration of the Sp divided by the R-R interval; AT-Sp = accelerating time of the Sp; Ac-Sp = acceleration of the Sp; TOSp = time from the onset of the QRS to the onset of the Sp; TPSp = time from the onset of the QRS to the peak of the Sp.

Results were expressed as mean ± SD with six data points collected in each analysis. The *t* values and the *P* values were given by comparing the results of the baseline with RVAP using paired Student’s *t* test. Statistical significance was defined as *P*<0.05. For *P* values <0.000, a value of <0.001 was chosen.

### Correlation Analysis

The duration of the Dp in the LAD correlated inversely with the TAoDP at baseline and RVAP (*r* = −0.860 and −0.945, *P*<0.05), and the acceleration of the Dp in the LAD correlated with the △P/T at baseline and RVAP (*r* = 0.917 and 0.848, *P*<0.05). The acceleration of the Sp in the AIV correlated with the △P/T at baseline (*r* = 0.914, *P*<0.05), and the peak velocity of the Sp in the AIV correlated with the AoSP at baseline and RVAP (*r* = 0.892 and 0.958, *P*<0.05). We could not establish other statistical significant linear correlations between the aortic pressure parameters and the Doppler hemodynamic parameters of the LAD and the AIV successfully in this study.

### Intraobserver and Interobserver Analysis

There was no statistically significant difference between the 2 observers or within an observer for the Doppler hemodynamic parameters of the LAD and the AIV (*P*>0.05). Intraobserver coefficient of variability was between 2.6% and 6.9%, with the smallest variation in measurements of the peak velocity of the Dp and the peak velocity of the Sp (2.6% and 2.9% respectively). The highest variation was found in D-Dp% and D-Sp% (6.9% and 6.5% respectively). Interobserver coefficient of variability was between 3.2% and 7.9%. The smallest variation was found in the peak velocity of the Dp and the peak velocity of the Sp (3.2% and 3.5% respectively). The highest variation was found in measurements of D-Sp% and D-Dp% (7.9% and 7.2% respectively).

## Discussion

Studies on the coronary venous system have been few, compared to those on the coronary arterial system, because of the technical difficulties of measurement [5]. In this experiment, we successfully quantified the effects of RVAP on hemodynamics of the LAD and AIV contrast to the baseline condition in open chest beagles using Doppler ultrasound imaging.

### Normal LV Electrical and Mechanical Activity and the Changes Induced by RVAP

Normal activation of the ventricles starts with conduction of the electrical impulse from the atrioventricular node to the His bundle, which on their turn divide first into a few major branches and subsequently into a network of subendocardially located Purkinje fibers. The impulse conduction of the myocardial muscular tissue in the LV occurs from apex to base and from endocardium to epicardium. The electrical impulse is conducted approximately four times faster in the Purkinje system than in the normal myocardium. As a result, all parts of the ventricular myocardium contract almost simultaneously, producing an efficient mechanical systole [6].

During RVAP, the conduction of the electrical wave front propagates through the myocardium, rather than through the His-Purkinje conduction system. The electrical wave front propagates more slowly and induces heterogeneity in electrical activation of the myocardium. This is characterized by a single breakthrough at the interventricular septum and the latest activation at the inferoposterior base of the LV. Similar to the changes in electrical activation of the ventricles, the mechanical activation pattern of the LV is changed during RVAP. Importantly, not only the onset of mechanical contraction is changed, but also the pattern of mechanical contraction. It has been demonstrated that the early activated regions near the pacing site exhibit rapid early systolic shortening, resulting in pre-stretch of the late-activated regions. As a result, these regions exhibit an increase in systolic shortening, imposing systolic stretch to the early activated regions exhibiting premature relaxation. This abnormal contraction pattern of the various regions of the LV may result in a redistribution of myocardial strain and work and subsequent less effective contraction. Both the abnormal electrical and mechanical activation pattern of the ventricles can result in changes in cardiac metabolism and perfusion, remodeling, hemodynamics, and mechanical function [7].

### The Phasic Blood Flow Patterns in the LAD and AIV at Baseline Condition

The blood flow pattern in the coronary circulation has a unique phasic nature: The distal coronary artery flow of the LAD is almost exclusively diastolic [8],[9], whereas venous outflow occurs predominantly during systole [10],[11]. With the spectral Doppler waveform analysis, we confirmed that the blood flow patterns in the LAD and AIV have such unique phasic natures. This phase opposition between the coronary artery and the vena coronaria can only be explained by the diastolic storage of arterial inflow in intramyocardial capacitance vessels and subsequent displacement into the coronary veins during ventricular contraction [12].

We found abrupt reverse of flow during early systole in the LAD in all cases at the baseline condition. Experimental study had demonstrated that small early systolic reverse flow (“slosh”) was detected in the peripheral coronary artery, such as the septal branch of normal subjects, and this reverse flow was caused by the milking effect produced by the contracting myocardium [13]. Iwakura et al. found that the flow pattern of the LAD in patients of acute myocardial infarction with the no-reflow phenomenon was characterized by the appearance of systolic retrograde flow [14]. However, they emphasized that the above-mentioned “slosh” can no longer be detected in the major epicardial coronary artery of healthy heart. Kajiya et al. investigated the phasic characteristics of normal LAD flow and velocity profiles during cardiac surgery: Among 10 cases, reverse flow was observed in early systole in 5 patients and in mid to late systole in 6 patients [15]. According with investigations of Kajiya et al. and the results of this study, we have to say that systolic retrograde flow in major epicardial coronary artery may be a physiological phenomenon that can appear in the healthy hearts.

Furthermore, transitory cessation flow during mid to late systole in the LAD and a momentary cessation flow at the end of diastole in the AIV at the baseline condition were recorded in all cases. Although we did our best to retrieve related literatures of pioneers, the phenomenon that the transitory stopped-flow in the LAD of normal hearts, could not be reappeared in their results. This maybe because of the different observational techniques were used, or because of the complex phasic blood flow patterns in coronary artery have not been recognized adequately in the past. Using quantitative angiography and casts of epicardial coronary veins in dogs, Hodgson et al. found that the appearance of gaps in the column of contrast material within the epicardial veins could be consistently seen, and the flow in epicardial coronary veins occurred as a bolus during each cardiac cycle [16]. The term vascular waterfall was used to describe the pressure-flow characteristics of collapsible vessels subjected to external compressive forces [17]. Uhlig et al. found that a vascular waterfall is present in epicardial coronary veins [18], which may be a possible explanation of the momentary cessation flow in the AIV of healthy hearts.

### Hemodynamic Changes in the LAD and AIV during RVAP

There were intimate correlations between ventricular asynchrony and coronary blood flow [19]. Studies have confirmed the existence of asynchronous mechanical activation sequence in the LV wall during RVAP [20],[21]. Using ventriculoatrial sequential pacing in dogs, Amitzur et al. found that the mid-right ventricular pacing was associated with a 10% reduction in the LAD blood flow when compared to the right atrial pacing [22]. Kolettis et al. reported that ventricular pacing was associated with a reduction in resting blood flow in LAD of normal human [23]. Our results showed no statistical significance in the peak velocity of the Dp in the LAD between each state, but the duration of the Dp shortened and the D-Dp% decreased. Compared with baseline condition, the peak velocity of the Sp in the AIV decreased, the duration of the Sp in the AIV shortened, and the D-Sp% decreased during RVAP. Abovementioned hemodynamic changes may be indirect reflections of blood flow reduction in the LAD and AIV during RVAP.

Left bundle branch block and RVAP have similar LV mechanical activation sequence [24]. Skalidis et al. found patients with left bundle branch block have an impairment of early diastolic blood flow in the LAD due to an increase in early diastolic compressive resistance resulting from delayed ventricular relaxation [25]. Youn et al. found the percentage of diastolic flow duration in LAD was significant shortened in the left bundle branch block group than the control group [26]. Combined with our results, we speculating that not only the RVAP induced LV mechanical activation sequence similar with that of the left bundle branch block, but also the hemodynamics in the LAD. The time from the QRS_O_ to the onset of the Dp in the LAD lengthened and the time from the QRS_O_ to the onset of the Sp in the AIV shortened during RVAP, these could be ascribed to the RVAP induced postponed mechanical relaxation and the early activated mechanical contraction in the LV wall [27].

Why RVAP induced a systolic negative wave S3 in the LAD at late systole, and induced a diastolic negative wave D1 in the AIV? RVAP might cause a threefold difference in regional myofibers work within the LV wall, and this difference appeared large enough to regard as an important determinant for abnormalities in myocardial perfusion during asynchronous electrical activation [27]. We speculating that the disturbed contraction activation sequence and the unbalanced contraction force in the LV wall during RVAP may be the determinant of such blood flow patterns in the LAD and the AIV. In all blood vessels, blood flows down a pressure gradient. Transient changes in flow in the coronary arteries induced by pressure gradients established between the proximal (aortic) and distal (intramyocardial) sites [28]. However, we could not measure the intramyocardial pressure and the right atria pressure synchronously in this pilot study. Therefore, the accurate mechanism of the phasic flow patterns in the LAD and AIV still need further elucidation in future studies.

### Practical Applications

Coronary flow velocity measurement by ultrasonic Doppler systems in laboratory and clinical research settings has provided information crucial to modern theories of coronary hemodynamics [29]. There is a natural need to research coronary circulation in normal as well as diseased conditions for clinical decision making. Physiologic pacing modes should maintain the naturally electrical and mechanical activity sequences, as well as the related unique physiologic blood flow patterns in the healthy heart as far as possible [30]. Considering the intimate correlation between ventricular mechanical activation sequence and coronary blood flow patterns, quantitative analysis of the hemodynamic characteristics in epicardial coronary arteries and vena coronaria may be used as a referred method for evaluation whether the pacing mode is more “physiologic” under clinical conditions.

Quantification of the blood flow patterns in the AIV with Doppler ultrasound is valuable for improving recognition of the hemodynamics of coronary venous system under various physiological or pathophysiologic conditions. In addition, we found the average distance between the LAD and the AIV was about 2.1 mm, and found no statistical significance between the Φ_LAD_ and the Φ_AIV_. The average distance between the LAD and the AIV of human hearts was about 3.8 mm [31]. These data indicating that we must be careful enough when detecting epicardial coronary arteries with transthoracic echocardiography under clinical conditions so as to avoid mistaking the concomitant vena coronaria as the target epicardial coronary arteries.

### Limitations

Clinical studies of coronary physiology will benefit greatly from combined measurements of coronary flow or velocity and pressure [32]. We have to say that measurement of the aortic pressure, the intramyocardial pressure and the right atria pressure synchronously are crucial important for accurate insight the physiology and pathophysiology of the coronary circulation. Rest Doppler velocities may be modulated by other factors, such as hypertension and microvascular impairment. Methodological limitations have hampered us to elucidate the accurate mechanism of the phasic blood flow patterns in the LAD and AIV. It is noteworthy that although the conspicuous hemodynamic changes in the LAD and AIV during RVAP were reflected uniquely and sensitively, but we did not provide direct measurement of myocardial mechanics. Therefore, the precise correlations between the ventricular asynchrony and the blood flow patterns in coronary circulation are real challenges for our further studies. To understand the chronic effects of RVAP on coronary circulation under clinical conditions, long term pacing animal model and large sample clinical studies should be included in future studies. We also stressed that these hearts are exposed with relief of the pericardial constraint, and a bipolar pacing electrode lead was implanted directly into the subepicardium of the right ventricular apical anterior wall. These may have undefined impacts, and limit the ability to extrapolate these findings to other situations where data are acquired noninvasively in un-instrumented hearts. One limitation in the analysis was the difference in heart rate between dogs in sinus rhythm and dogs during RVAP. Therefore, direct His bundle pacing or atrial pacing [33],[34] with fixed pacing rate at 160 bpm should be considered as a control group in our further studies. And the disturbance of the low frequency high intensity Doppler signals in the spectral Doppler waveforms, which resulted from the movement of the vessel and the perivascular tissues, deserves better regulation in future studies.

### Conclusions

Obvious hemodynamic changes in the LAD and AIV during RVAP have been quantified by spectral Doppler ultrasound. Healthy hearts with normal sinus rhythm maintains its concordant myocardial contraction and relaxation, and the characteristics of the blood flow patterns in the coronary circulation may be taken as normal references. In contrast, the abnormal blood flow patterns in the epicardial coronary arteries and vena coronaria may be sensitive and important hints of the disturbed cardiac electrical and mechanical activity sequences. It will be of practical importance to elucidate the accurate relationships between the blood flow patterns of the coronary circulation and the myocardial mechanical activation sequence in further studies, so as to determine whether spectral Doppler waveform analysis of the epicardial coronary circulation could be incorporated effectively into clinical practice.
